# Novel Therapies for Aggressive B-Cell Lymphoma

**DOI:** 10.1155/2012/302570

**Published:** 2012-03-25

**Authors:** Kenneth A. Foon, Kenichi Takeshita, Pier L. Zinzani

**Affiliations:** ^1^Celgene Corporation, 86 Morris Avenue, Summit, NJ 07901, USA; ^2^Department of Hematology and Oncological Sciences “L. e A. Seràgnoli”, University of Bologna, Via Massarenti, 9-40138 Bologna, Italy

## Abstract

Aggressive B-cell lymphoma (BCL) comprises a heterogeneous group of malignancies, including diffuse large B-cell lymphoma (DLBCL), Burkitt lymphoma, and mantle cell lymphoma (MCL). DLBCL, with its 3 subtypes, is the most common type of lymphoma. Advances in chemoimmunotherapy have substantially improved disease control. However, depending on the subtype, patients with DLBCL still exhibit substantially different survival rates. In MCL, a mature B-cell lymphoma, the addition of rituximab to conventional chemotherapy regimens has increased response rates, but not survival. Burkitt lymphoma, the most aggressive BCL, is characterized by a high proliferative index and requires more intensive chemotherapy regimens than DLBCL. Hence, there is a need for more effective therapies for all three diseases. Increased understanding of the molecular features of aggressive BCL has led to the development of a range of novel therapies, many of which target the tumor in a tailored manner and are summarized in this paper.

## 1. Introduction

Many variations of aggressive B-cell lymphoma (BCL) exist, each with distinct molecular, biological, and cytogenetic characteristics [1]. Examples include diffuse large B-cell lymphoma (DLBCL), Burkitt lymphoma, and mantle cell lymphoma (MCL). Malignant lymphomas can arise at multiple stages of normal B-cell development, with the germinal center serving as the probable origin of many types of lymphoma [2]. In the germinal-center reaction, mature B cells are activated by antigen, in conjunction with signals from T cells. During this process, B-cell DNA is modified, which results in an altered B-cell receptor. These genetic modifications are prerequisite to a normal immune response but are also the source of genetic defects that result in accumulated molecular alterations during the lymphomagenesis process [3–5].

DLBCL is the most common lymphoid malignancy, accounting for approximately 25 to 30% of all adult lymphomas in the western world [6]. Chemoimmunotherapy with rituximab plus anthracycline-based combination regimens has substantially improved long-term disease control, with more than 50% of patients still in remission 5 years after treatment [7–10]. There are 3 histologically indistinguishable molecular subtypes of DLBCL: the activated B-cell-like (ABC) subtype, the germinal-center B-cell-like (GCB) subtype, and primary mediastinal BCL (PMBL) [11–13]. These subtypes differ in terms of gene expression [13, 14] and are believed to originate in B cells at different stages of differentiation [15]. In addition, the process of malignant transformation differs for each subtype, resulting in distinctive patterns of genetic abnormality [11, 15]. Clinical presentation and responsiveness to targeted therapies also vary across the subtypes.

Gene expression in GCB lymphomas is characteristic for germinal-center B cells [11, 15, 16], with, for example, deletion of the tumor suppressor gene *PTEN *[17], and *p53* mutations [18] being specific to GCB lymphomas. Genetic abnormalities that are characteristic for ABC DLBCL include, for example, deletion of the *INK4*α*/ARF* tumor suppressor locus on chromosome 9 and amplification of a 9-Mb region on chromosome 19 [19]. Loss of these tumor suppressors impedes the action of chemotherapy and may contribute to the poor prognosis associated with this subtype. PMBL, although not easily differentiated clinically from other lymphoma subtypes, is readily distinguishable by gene-expression profiling [12, 13] such as deletion of *SOCS1*, a suppressor of JAK signaling [20–22].

Burkitt lymphoma, an aggressive BCL characterized by a high degree of proliferation of the malignant cells and deregulation of the *MYC *gene, relies on morphologic findings, immunophenotyping results, and cytogenetic features for diagnosis [2]. However, DLBCL and Burkitt lymphoma can have overlapping morphologic and immunophenotypic features, and the characteristic t(8;14) translocation found in Burkitt lymphoma also occurs in ≤15% of DLBCL cases [23]. While the regimen of rituximab, cyclophosphamide, hydroxydaunorubicin, vincristine, and prednisone (R-CHOP) is typically used as a first-line treatment for DLBCL, Burkitt lymphoma requires more intensive chemotherapy regimens [24]. 

MCL, a mature B-cell lymphoma, is almost invariably associated with the t(11;14) translocation with overexpression of cyclin D1 [25]. Several morphologic variants exist, some of which are predictive of a poorer prognosis [26]. Deletions of the *INK4*α*/ARF* locus on chromosome 9p21 [27] and mutations of *p53* in 17p13, for instance, are also associated with a more aggressive histology [27–29].

Significant progress has been made in the management of patients with aggressive DLBCL. Addition of rituximab to the CHOP regimen (R-CHOP) [30] has resulted in fewer patients with disease progression. However, recent trial results have provided no evidence to indicate that rituximab combined with CHOP given every 14 days (R-CHOP14) improves overall survival (OS) or progression-free survival (PFS) compared with the standard regimen of R-CHOP given every 21 days (R-CHOP21) in newly diagnosed DLBCL [31]. 

Consequently, a substantial unmet need still exists. Depending on the DLBCL subtype, patients experience significantly different survival rates following chemotherapy, with the ABC subtype in particular being associated with a poorer outcome [11, 19, 32]. Recurrent disease, especially after rituximab exposure, is also a concern, and patients with early relapse after rituximab-containing first-line therapy have been shown to have a poor prognosis [33]. In MCL, the addition of rituximab to conventional chemotherapy regimens has increased overall response rates (ORRs), but not OS compared with chemotherapy alone [34]. 

As we further our understanding of the molecular characteristics of aggressive BCL, we hope it will lead to the design of therapies that target the tumor and its microenvironment more directly and more effectively.

## 2. Cytotoxic Therapies

Several new cytotoxic agents are being investigated for the treatment of aggressive lymphomas (Table 1). Bendamustine has shown single-agent and combination activity in indolent lymphomas [35–37]. Although approved for this indication in some countries, evidence supporting its use in treating aggressive lymphomas has been limited. Recently, a feasibility and pharmacokinetic study of bendamustine in combination with rituximab in relapsed or refractory (R/R) aggressive B-cell non-Hodgkin lymphoma (NHL) confirmed that bendamustine 120 mg/m^2^ plus rituximab 375 mg/m^2^ was feasible and well tolerated and showed promising efficacy [38]. A subsequent phase II study of bendamustine as monotherapy showed a 100% ORR and a 73% complete response (CR) in R/R MCL patients [39]. Preliminary data of another study of bendamustine in combination with rituximab in elderly patients with R/R DLBCL demonstrated an ORR of 52% [40]. A phase III study of this combination showed better efficacy than a fludarabine-rituximab combination in patients with relapsed follicular, other indolent NHLs and MCL [41]. In another phase III study in previously untreated indolent BCL and MCL patients, the bendamustine-rituximab regimen was superior to R-CHOP in terms of CR and PFS [42]. Retrospective analyses of clinical use in Italy [43] and Spain [44] have indicated that treatment with bendamustine alone, or in combination with rituximab, is efficacious and has an acceptable safety profile in heavily pretreated NHL and chronic lymphocytic leukemia (CLL) patients. The most common adverse events associated with bendamustine were hematologic or gastrointestinal in nature and mild to moderate in intensity.

The activity profile of the gemcitabine-oxaliplatin (GEMOX) combination makes it an attractive regimen for use as salvage therapy for several types of lymphoma. Phase II studies have demonstrated significant activity of GEMOX in combination with rituximab (GEMOX-R) in R/R DLBCL [45] and MCL [46]. The major toxicities observed with this regimen were grade 3 or 4 neutropenia and thrombocytopenia. Promising activity with acceptable toxicity has been shown for GEMOX-R in patients with R/R B-cell NHL who are ineligible for high-dose therapy [47] or subsequent transplant [48]. A phase III trial of the novel aza-anthracenedione pixantrone dimaleate [49] was prompted by the absence of reliable durable efficacy in patients with aggressive NHL who have relapsed following multiple lines of therapy. This trial showed superior efficacy compared with a number of alternative third-line single-agent therapies. Neutropenia and leukopenia were the most common grade 3 or 4 adverse events. A second phase III trial, comparing pixantrone-rituximab with gemcitabine-rituximab in patients with R/R DLBCL that are not eligible for stem cell transplantation (SCT), is currently recruiting (NCT01321541). A liposomal formulation of vincristine has also shown activity in patients with aggressive NHL that have relapsed after second-line therapy [50]; grade 3 or 4 neurotoxicity occurred in 32% of patients.

Other novel agents target mitotic spindle proteins; Eg5, for example, has emerged as a unique mitotic spindle target [51]. SB-743921 is a novel kinesin spindle protein inhibitor that has shown significant activity in both *in vivo* and *in vitro* models of aggressive DLBCL. In a phase I/II dose-finding study, activity was observed in heavily pretreated NHL and Hodgkin lymphoma (HL) patients, with neutropenia (47%) reported as the most frequent grade 3 or 4 toxicity [52].

Clofarabine is a second-generation purine analog approved by the United States Food and Drug Administration (FDA) for intravenous use in R/R pediatric acute lymphoblastic leukemia (ALL). Purine analogs demonstrate significant clinical activity in NHL, with a phase I preliminary evaluation of an oral formulation of clofarabine in relapsed or refractory NHL reporting an ORR of 35%, with no grade 3 or 4 nonhematologic toxicities [53].

## 3. Antibodies

### 3.1. Anti-CD20 Monoclonal Antibodies (mAbs) (Table 2)

The chimeric anti-CD20 mAb rituximab improved therapeutic outcomes considerably for patients with B-cell malignancies, particularly when combined with chemotherapy [54]. However, resistance and reduced response to retreatment led to the development of second-generation humanized (or primarily human) mAbs, which have greater cytotoxicity and stronger direct effects on B cells.

Veltuzumab is a humanized CD20 mAb with complementarity-determining regions differing from rituximab by only 1 amino acid, a characteristic believed to account for the markedly reduced off-rates demonstrated by veltuzumab compared with rituximab [55]. A major response was demonstrated in a phase I/II dose-escalation trial in patients with R/R NHL, with no evidence of immunogenicity [56]. B-cell depletion was observed from first infusion, even at the lowest dose of 80 mg/m^2^. Adverse events were transient, mild to moderate, and occurred mostly at first infusion, a notable finding given the short infusion times. A phase I study with veltuzumab in combination with the anti-CD74 antibody milatuzumab in patients with R/R NHL is ongoing [57].

The fully human CD20 mAb, ofatumumab, has been FDA-approved for the treatment of fludarabine- and alemtuzumab-refractory CLL [58] and is currently being evaluated in NHL. Ofatumumab induces B-cell depletion via mechanisms similar to rituximab, but with substantially more complement-dependent cytotoxicity. Recent *in vivo* data suggest ofatumumab may be more potent than rituximab in both rituximab-sensitive and rituximab-resistant models and may potentiate the antitumor activity of chemotherapy agents commonly used in the treatment of B-cell NHL [59]. Initial results from a phase II study in relapsed or progressive DLBCL showed that single-agent ofatumumab is well-tolerated with evidence of efficacy [60]. In this patient population, response to the last systemic treatment appeared to influence response to ofatumumab; a subsequent study of ofatumumab in combination with ifosfamide, carboplatin, etoposide (ICE) or dexamethasone, Ara-C, and cisplatin (DHAP) chemotherapy regimens (NCT00823719) is ongoing.

GA101 is a novel humanized CD20 mAb that binds CD20 in a manner completely different to that of rituximab and ofatumumab [61]. In preclinical studies it has demonstrated superior efficacy compared with both agents [62, 63], and an initial phase I trial with dosing every three weeks demonstrated promising activity with no dose-limiting toxicity (DLT) [64]. A second dose-finding study in patients with R/R NHL (*n* = 22; ORR: 25% at 3 months) [65] has been followed by a phase II study in heavily pretreated patients with R/R DLBCL and MCL. Treatment was well tolerated, and promising evidence of efficacy was shown [66]. Recent *in vivo* studies have shown enhanced inhibition of tumor growth for GA101 in combination with bendamustine, fludarabine, and the B-cell lymphoma 2 (Bcl-2) family inhibitors ABT-737 and ABT-263 [67].

### 3.2. Novel Targeted mAbs (Table 2)

The humanized mAb, epratuzumab, targets CD22 which is a B-cell marker thought to play a role in B-cell activation, cell-surface receptor circulation, and modulation of antigen-receptor signaling [68]. In a phase II trial in patients with R/R NHL, the combination of epratuzumab and rituximab resulted in considerable ORRs in both follicular lymphoma and DLBCL [69]. In a subsequent phase II study, in which epratuzumab was added to R-CHOP as first-line therapy for DLBCL, an ORR of 95% was reported. Substantial responses were documented even when patients were separated into low- and high-risk international prognostic index (IPI) groups [70]. Positron emission tomography (PET) scan data confirmed a functional CR rate of 87% in this study, with attainment of PET negativity by completion of therapy being associated with a good outcome [71].

Milatuzumab is a humanized anti-CD74 mAb in clinical evaluation for the treatment of multiple myeloma (MM), CLL, and NHL. In preclinical trials, milatuzumab monotherapy has demonstrated therapeutic activity against various B-cell malignancies, while the addition of milatuzumab to numerous agents including rituximab and fludarabine enhanced the therapeutic efficacy in a variety of B-cell malignancy cell lines [72]. As milatuzumab combined with rituximab was shown to cause MCL cell death [73], further evaluation of this combination in MCL is warranted. A dose-escalation study of a milatuzumab-veltuzumab regimen in R/R NHL is ongoing [57].

Lucatumumab (HCD122), a mAb that is a pure antagonist of the CD40 transmembrane receptor, has been evaluated clinically in CLL and MM and is currently under evaluation in a variety of lymphomas, including DLBCL and MCL [74]. Initial efficacy has been shown in an ongoing phase Ia/II trial in patients who had progressed after multiple prior therapies, with DLTs limited to clinically asymptomatic and reversible grade 3 or 4 elevations of amylase and/or lipase and grade 3 or 4 elevations of alanine aminotransferase (ALT) and/or aspartate aminotransferase (AST). 

The humanized anti-CD40 mAb, dacetuzumab (SGN-40), has demonstrated antiproliferative and apoptotic activity against a panel of high-grade BCL cell lines [75]. Dacetuzumab was shown to enhance the antitumor activity of rituximab in NHL cell lines and xenograft models, suggesting that antibody-mediated signaling through both CD20 and CD40 may be an effective strategy in the treatment of NHL [76]. Dacetuzumab in combination with rituximab and gemcitabine for the treatment of NHL is currently being evaluated in a phase Ib study [77].

Small modular immunopharmaceuticals (SMIPs) are single-polypeptide chains consisting of a single-chain Fv linked to human IgG hinge, CH2, and CH3 domains [78]. TRU-016, a novel humanized anti-CD37 SMIP protein, has demonstrated single-agent activity as well as synergy with bendamustine, rituximab, rapamycin, and temsirolimus and an additive benefit with doxorubicin [79–81]. TRU-016 is currently being evaluated in a phase I study in relapsed NHL and CLL (NCT00614042).

### 3.3. Bispecific Antibodies (BsAbs)

New mAbs are being tested in combination with rituximab, including BsAbs that target CD20 and CD22 simultaneously [82]. HB22.7 is an anti-CD22 mAb that specifically blocks the interaction of CD22 with its ligand, has direct cytotoxic effects, and initiates CD22-mediated signal transduction. The cell binding, signaling patterns, and lymphomacidal activity of a BsAb (Bs20x22) combining rituximab and HB22.7 have been evaluated using a xenograft model of human NHL. Efficacy was demonstrated by *in vitro *cytotoxicity and apoptosis assays, p38 activation, and xenograft models. Bs20x22 appeared to be more efficacious than the combination of rituximab and HB22.7 and eliminated the need for sequential administration of 2 separate mAbs. 

The recent creation of an anti-CD20/human leukocyte antigen (HLA)-DR-interferon- (IFN-) *α*2*β* BsAb immunocytokine (designated 20-C2-2*β*) is expected to have greater *in vivo* potency than IFN-*α* due to improved pharmacokinetics and targeting specificity and may potentially be useful in a variety of hematopoietic tumors that express either CD20 or HLA-DR [83].

Bispecific T-cell engager molecules (BiTEs) are antibodies that target both an antigen on malignant cells and CD3 on the surface of T cells [84]. In a phase I trial in relapsed NHL, the anti-CD19/CD3 BiTE antibody, blinatumomab, produced multiple responses in 52 patients. Implementation of a double-step dose-escalation procedure avoided treatment discontinuations due to CNS events [85].

Recently, preclinical data have been presented for a number of other agents, including anti-HLA-DR humanized mAb IMMU-114 [86], anti-CD47 antibody [87], anti-CD137 antibody [88], and the anti-CD19 mAb XmAb5574 [89].

### 3.4. Antibody-Drug Conjugates (ADCs) (Table 3)

ADCs are mAbs attached to cytotoxic drugs via chemical linkers [90]. Inotuzumab ozogamicin (CMC544) is composed of the anti-CD22 antibody inotuzumab and calicheamicin, a cytotoxic agent derived from the bacteria *Micromonospora echinospora*, which acts by cleaving DNA [91]. A phase I trial with 48 patients with R/R lymphoma showed ORRs of 69% and 33% for follicular lymphoma and DLBCL, respectively [92]. Inotuzumab ozogamicin was well tolerated; the most frequent adverse event was thrombocytopenia, which occurred at grade 3 or 4 in 57% of patients. In a phase I/II trial where inotuzumab was combined with rituximab in patients with relapsed follicular lymphoma or DLBCL, the response rates and 6-month PFS were 88% and 100% for follicular lymphoma and 71% and 66% for DLBCL, respectively [93]. Recently, preliminary results from a trial of inotuzumab plus rituximab in relapsed DLBCL patients followed by SCT were reported [94]. A best ORR of 21% was observed, with no new safety concerns. The inotuzumab-rituximab combination was also used in a study in Japanese patients with R/R B-cell NHL, resulting in an ORR of 80%; adverse events leading to discontinuation included neutropenia and hyperbilirubinemia [95]. Further studies of this combination in NHL are ongoing (NCT00299494; NCT01232556).

^90^Y-epratuzumab-tetraxetan is a radiolabeled, humanized anti-CD22 antibody that has been used for fractionated radioimmunotherapy (RIT) and has shown high rates of durable CRs with manageable hematologic toxicity in previously treated patients with indolent and aggressive NHL [96]. A phase II study, currently underway, is assessing ^90^Y-epratuzumab-tetraxetan as consolidation therapy after first-line chemotherapy in disseminated DLBCL patients over 60 years of age [97]. 31% of patients in whom a CR, unconfirmed CR, or worse, was reported with R-CHOP improved their remission status 6 weeks after RIT. The common grade 3 or 4 toxicities reported were neutropenia (78%) and thrombocytopenia (74%). A phase I/II study of ^90^Y-epratuzumab-tetraxetan combined with veltuzumab in patients with R/R aggressive NHL is currently recruiting (NCT01101581). Preclinical data indicate that the efficacy of epratuzumab conjugated with SN-38 (the active component of the topoisomerase I inhibitor, irinotecan) may potentially be enhanced when combined with the CD20 immunotherapeutic, veltuzumab [98].

^90^Y-ibritumomab tiuxetan (^90^Y-IT), an anti-CD20 murine antibody linked to a beta-emitting isotope, is approved for use in indolent lymphoma [99]. In a phase II trial, ^90^Y-IT induction followed by rituximab maintenance in patients with R/R DLBCL had an acceptable toxicity profile and the 2-week outpatient ^90^Y-IT infusion produced response rates and durations similar to those of more prolonged cytotoxic chemotherapy regimens. Another phase II trial showed 6 cycles of fludarabine and mitoxantrone (FM) followed by ^90^Y-IT in previously untreated, indolent, nonfollicular NHL to be tolerable and effective, with a CR rate of 50% after FM chemotherapy increasing to 100% at the end of the treatment regimen [100]. The Eastern Cooperative Oncology Group (ECOG) carried out a phase II study of R-CHOP followed by ^90^Y-IT in previously untreated MCL. This trial showed that failure-free survival appeared prolonged over that expected with R-CHOP alone and the regimen was considered to be safe, with neutropenia and thrombocytopenia being the most frequent adverse events [101].

Consolidative RIT with iodine-131 tositumomab was administered in a phase II trial in 86 patients with previously untreated DLBCL [102]. In this trial, 5 patients died of toxicities possibly related to therapy, including 1 case of febrile neutropenia, 1 case of acute myeloid leukemia (AML), and one case of renal failure; 2 deaths were caused by cardiac ischemia, 1 of which occurred after a gastrointestinal bleed in a patient that became thrombocytopenic after iodine-131 tositumomab. The 1-year PFS and OS estimates were 75% and 83%, respectively; given that the estimated historical 1-year PFS rate with R-CHOP alone in this population is 74%, a consolidation strategy utilizing iodine-131 tositumomab after 8 cycles of CHOP (6 with rituximab) for DLBCL does not appear to be promising in regard to 1-year PFS or OS. The authors concluded that in this population of DLBCL, early progressions, deaths, and declining performance status during CHOP limit the number of patients who can ultimately benefit from a planned consolidation approach. The use of novel agents earlier in therapy may have a greater impact in DLBCL than consolidation or maintenance approaches [102]. A phase II study of iodine-131 tositumomab for 1st- or 2nd-relapse indolent BCLs, or BCLs that have transformed to a more aggressive histology, has been completed recently (NCT00950755).

The binding properties, internalization kinetics, and clinicopathological activity of the ADC, brentuximab vedotin (SGN-35), were described recently [103]. In a phase 1, weekly dosing study, brentuximab induced multiple objective responses in patients with R/R CD30-positive lymphomas [104]. DLTs included diarrhea, vomiting, and hyperglycemia.

A novel ribonuclease-based immunotoxin comprising quadruple ranpirnase (Rap) site specifically conjugated to an anti-CD22 IgG has shown potent antilymphoma activity in *in vivo* and *in vitro* assays [105].

## 4. Additional Novel Strategies

Adoptive transfer of autologous T-cells expressing anti-CD19 chimeric antigen receptors (CARs) is a potential new approach for treating B-cell malignancies [106]. A phase I clinical trial of B-cell malignancies treated with autologous anti-CD19-CAR-transduced T cells is ongoing, with data published on five patients, having received two doses of cyclophosphamide 60 mg/kg and five doses of fludarabine 25 mg/m^2^ followed by infusions of anti-CD19-CAR-transduced T cells and administration of high-dose interleukin-2 (IL-2). Initial results appear promising.

Therapeutic vaccination holds enormous potential as a complementary treatment for NHL, and IL-2 has a wide range of immunologic effects and is able to induce regression of metastatic human tumors [107]. In a preclinical study, a therapeutic vaccine using tumor cells activated by *Salmonella *infection and IL-2 has been shown to induce antitumor immunity in BCL. This approach may have therapeutic value in promoting systemic immunity against human NHL.

To circumvent cytotoxic T-lymphocyte (CTL) tolerance of tumor-associated antigens, noncognate cytotoxic T cells have been retargeted against CD20^+^ tumor cells using [Fab' x MHC class I/peptide] conjugates. The ability of [Fab' x MHC class I/peptide] constructs to cause proliferation of OT-1 cells (a transgenic T-cell receptor model in which the CD8+ T-cells express a T-cell receptor specific for the SIINFEKL peptide of ovalbumin) *in vitro *suggests that it may be possible to use a single molecule to generate a secondary cytotoxic T-cell response and, subsequently, to retarget it, thus increasing the feasibility of the approach if adopted in the clinical setting [108].

## 5. Other Targeted Therapies

### 5.1. Immunomodulating Agents (Table 4)

Thalidomide and its newer derivative, lenalidomide, have multifaceted antitumor effects that include immunomodulatory effects via natural-killer-cell recruitment and cytokine modulation, antiangiogenesis, and the ability to alter tumor and stromal-cell interactions [109]. An early study of thalidomide plus rituximab found responses in 13/16 patients with relapsed MCL, although follow-up was limited [110]. More recently, data from 58 patients in a French compassionate-use study provided good response data with limited toxicity [111]. Lenalidomide monotherapy was evaluated in a phase II study of 49 patients with R/R aggressive NHL, including 15 with MCL [112, 113], and demonstrated an ORR of 35% with a median duration of response (DR) of 6.2 months. Cytopenias, fatigue, constipation or diarrhea, rash, and fever were common adverse events. A larger, international, confirmatory phase II study in patients with R/R DLBCL or MCL showed an ORR of 35%. Adverse events included grade 3 or 4 neutropenia (41%) and thrombocytopenia (19%) [114]. Pooled data of patients who had received prior SCT from these 2 studies suggest lenalidomide to be efficacious, with an ORR of 39%, and well tolerated [115].

Preclinical evidence for synergistic activity of the lenalidomide-rituximab combination in MCL [116, 117] is supported by results of a phase I/II study, which has shown a 53% ORR in patients with R/R MCL. Grade 3 or 4 toxicities included neutropenia (37 events in 45 patients) [118]. The evolving role of lenalidomide in relapsed MCL is further strengthened by data from a phase II trial of lenalidomide in combination with dexamethasone (ORR: 55%; grade 3 or 4 neutropenia in 48% of patients) [119], and with rituximab and dexamethasone (ORR: 57%) [120]. Lenalidomide is also being evaluated in combination with R-CHOP (R2-CHOP) in a phase I/II trial in patients with aggressive BCLs [121]. A second phase I study is ongoing [122]. Interim analysis of a phase I/II trial of lenalidomide plus R-CHOP21 showed multiple CRs and moderate hematologic toxicity (grade 3 or 4 neutropenia: 28%) [123]. Recruitment is ongoing for a phase I/II study of lenalidomide, rituximab, and bendamustine in aggressive BCL (NCT00987493).

### 5.2. Proteosome Inhibitors (Table 5)

Bortezomib, a reversible inhibitor of the chymotrypsin-like activity of the 26S proteasome, disrupts normal homeostatic mechanisms in cells [124]. This agent is used widely to treat MM and is now also approved for use in MCL. Its activity in combination with other agents has been investigated in several recent studies. R-CHOP plus bortezomib produced an ORR of 91% in previously untreated MCL patients, with neutropenia (23%) and thrombocytopenia (14%) among the grade 3 or 4 cytopenias that were reported [125]. A phase II study of bortezomib in combination with bendamustine and rituximab in patients with R/R indolent and MCL produced an ORR of 84%, although the triple regimen appeared to be more toxic than the bendamustine-rituximab regimen alone [126]. Interim data from a phase II study suggested promising results for a regimen of bortezomib plus dose-dense CHOP every 2 weeks as first-line treatment in disseminated DLBCL [127]. A recent study by Dunleavy and colleagues [128] showed that although bortezomib alone had no activity in DLBCL, when combined with chemotherapy it demonstrated a significantly higher response in ABC compared with GCB DLBCL. These results indicate that bortezomib specifically benefits non-GCB DLBCL patients, who normally exhibit inferior outcomes relative to GCB subtype patients after therapy with CHOP or R-CHOP. An ongoing phase II study of R-CHOP with or without bortezomib is prospectively enrolling only those patients with the non-GCB subtype DLBCL [129].

The combination of bortezomib and rituximab in a weekly schedule has been shown to be effective with little hematologic toxicity in a phase II study in R/R indolent BCL and MCL [130]. In another phase II study, a combination of bortezomib plus rituximab, doxorubicin, dexamethasone, and chlorambucil (RiPAD+C) was shown to be feasible and well tolerated as a first-line therapy in elderly MCL patients [131]. Bortezomib was used in place of vincristine in the standard rituximab, cyclophosphamide, vincristine, and prednisone (R-CVP) regimen in a phase I trial in R/R indolent DLBCL and MCL [132]. The R-CBorP regimen appeared to be well tolerated and the efficacy data looked promising. Several other phase I studies are further exploring potential uses of bortezomib, with positive data reported for its use in combination with conatumumab [133], gemcitabine [134], and ^90^Y-IT [135].

Numerous trials that are ongoing or recruiting, are investigating the combination of bortezomib with rituximab-ICE (RICE) (NCT01226849; NCT00515138), tositumomab (NCT00398762), and vorinostat (NCT00837174). Preclinical data support further combination regimens, including romidepsin [136], autophagy inhibitors [137], the murine double minute (MDM2) inhibitor, nutlin-3 [138], and the BH3 mimetic, obatoclax [139].

NPI-0052 is a proteasome inhibitor with a novel bicyclic structure [140]. In a phase I study, NPI-0052 produced dose-dependent pharmacologic effects, with less peripheral neuropathy, neutropenia, and thrombocytopenia than was typically noted with other proteasome inhibitors. MLN9708 has shown activity in preclinical models of lymphoma [141, 142]. Further, the novel proteasome inhibitor carfilzomib has been shown to interact synergistically with histone deacetylase inhibitors (HDACIs) [143].

### 5.3. Phosphatidylinositol 3-Kinase (PI3K) Pathway (Table 5)

The PI3K-signaling pathway plays a major role in regulating cell growth and survival and is often deregulated as a result of the mutation or amplification of Akt [144, 145]. The mammalian target of rapamycin (mTOR) kinase is an essential mediator of growth signaling that originates from PI3K. mTOR activation by Akt leads to cell proliferation and survival by modulating critical molecules such as cyclin D1.

The rapamycin analogs, everolimus (RAD001) and temsirolimus, are approved by the FDA for renal cell carcinoma and have demonstrated activity against lymphoma cells both *in vitro* and *in vivo* [146, 147]. Everolimus was evaluated in a single-agent phase II study in patients with relapsed aggressive NHL in whom standard therapy failed [148]. Significant responses were noted; grade 3 or 4 events included anemia (14%), neutropenia (18%), and thrombocytopenia (38%). In another single-agent phase II study, everolimus showed moderate activity in patients with R/R MCL; grade 3 or 4 anemia and thrombocytopenia were reported in 11% of patients [149]. A phase II study of the combination of everolimus and rituximab in R/R DLBCL has just been completed (NCT00869999). Preliminary results from a phase II study in MCL patients refractory to bortezomib reported promising single-agent activity and good tolerability [150]. A Japanese phase I study in patients with R/R NHL has also shown preliminary evidence of activity of everolimus in NHL [151]. Phase I/II studies exploring the novel combinations of everolimus and panobinostat (LBH589) [152] or bortezomib (NCT00671112) are ongoing.

A phase III study of R/R MCL comparing temsirolimus with physician's choice demonstrated an ORR of 22% and 2%, respectively [153]. A phase II study of temsirolimus plus rituximab produced a 59% ORR; the most common grade 3 or 4 adverse event in rituximab-sensitive and -refractory patients was thrombocytopenia (17% and 38%, resp.) [154]. Temsirolimus also shows some activity in DLBCL with an ORR of 28%, a CR of 12%, and a median PFS of 2.6 months [155].

The PI3K p110*δ* isoform is preferentially expressed in cells of hematologic origin and in a variety of malignant cells [156]. CAL-101 is a potent p110*δ* inhibitor and has shown acceptable safety and promising pharmacodynamic and clinical activity in a variety of hematologic malignancies, as a single agent [157–159] and in combination with rituximab or bendamustine [160].

SF1126 is a dual PI3K/mTOR inhibitor and is currently in phase I development in B-cell malignancies [161]. Other novel approaches under investigation in preclinical trials include combining mTOR inhibitors with rapamycin-resistant T cells [162], targeting the PI3K/Akt/survivin pathway with the protease inhibitor, ritonavir [163], dual mTORC1/mTORC2 inhibition [164], and use of immunosuppressive agents (e.g., fingolimod; FTY720) to downregulate cyclin D1 and pAkt [165].

### 5.4. DACs/HDACIs (Table 5)

Several groups of HDACIs have been developed, and they all show activity in lymphoma, mostly cutaneous [166]. HDACIs have been shown to promote apoptosis and to reduce angiogenesis. Vorinostat, registered for R/R cutaneous T-cell lymphoma (CTCL), works synergistically with other drugs, but its role in the treatment of DLBCL is not clear yet. A number of phase I studies of vorinostat-combination regimens in relapsed lymphoma are either ongoing or have been completed recently. These studies have incorporated R-ICE/ICE [167], pegylated liposomal doxorubicin [168], and conatumumab [133]. Preclinical evidence supporting the clinical development of vorinostat plus the novel Aurora kinase inhibitor, MK-5108, has also been presented [169]. A recent safety and tolerability analysis of prior phase I and II trials of vorinostat-based therapy in CTCL, other hematologic malignancies, and solid tumors, highlighted fatigue (62%) and nausea (56%) as the most common drug-associated adverse events, with fatigue (12%) and thrombocytopenia (11%) the most common grade 3 or 4 adverse events [170].

Valproic acid functions as a HDACI, although data on its activity are limited [171]. A recent phase II trial in refractory lymphoma produced 4/14 responses (all partial responses (PRs)). An earlier phase I study with decitabine showed dose-limiting myelosuppression and infectious complications which precluded dose escalation to a minimum effective dose [172].

Panobinostat is an oral pan-DACI that has shown activity in a variety of cancers. Responses have been documented in a phase II study in relapsed HL [173] and in combination with everolimus in a phase I/II study in R/R HL and NHL [152]. It is also being investigated in DLBCL, where preclinical activity has been observed in combination with decitabine [174].

The HDACI, belinostat, has broad preclinical activity [175]. Interim results from a phase I study in patients with lymphoid malignancies provided evidence of tumor shrinkage, and a phase II, Southwest Oncology Group (SWOG) study in patients with R/R aggressive B-cell NHL is ongoing (NCT00303953).

PCI-24781 is a broad-spectrum HDACI, which has shown activity in lymphoma cell lines and models [176]. It has also demonstrated safety and initial clinical benefit in a phase I study in R/R lymphoma. 

Entinostat (SNDX-275) is an oral, class I isoform-selective HDACI [177]. A number of responses have been observed in an ongoing phase II study in R/R NHL, and synergistic preclinical activity has been reported in combination with bortezomib [178].

Preclinical activity has also been observed with panobinostat [179, 180] and the oral heat-shock-protein- (Hsp-) 90 inhibitor, SNX-2112 (in combination with bortezomib and rituximab) [181].

### 5.5. Cell Death (Bcl Family) (Table 5)

The intrinsic cell-death pathway is triggered at the mitochondria by a range of signals, with the most important regulators residing in the Bcl-2 family [182]. The Bcl-2 antisense nucleotide, oblimersen, was evaluated in a phase II study in combination with rituximab in patients with recurrent B-cell NHL. An ORR of 42% was found and most toxicity was low in grade and was reversible [183].

ABT-263 (navitoclax) is currently being investigated in clinical trials of lymphoma, as monotherapy [184] and in combination with rituximab [185]. The experimental Bcl-2 inhibitor, ABT-737, is in preclinical development for MCL [186] and DLBCL [67]. Other agents in preclinical development include obatoclax (in combination with bortezomib) [140, 187] and YM155 [188, 189].

### 5.6. Kinase Inhibitors (Table 6)

Aurora kinases A and B (AAK and ABK) are oncogenic serine/threonine (S/T) kinases that play central roles in the mitotic phase of the eukaryotic cell cycle [190]. Overexpression of Aurora kinases during the cell cycle can override mitotic and spindle check-points leading to aneuploidy in many human cancers. Gene expression profiling in aggressive B- and T-cell NHL has shown the Aurora kinases to be overexpressed suggesting that they may be key component genes of the “proliferative” signature.

MLN8237 is a selective AAK inhibitor, which showed synergy with docetaxel in preclinical models of MCL [191]. In a phase I study in patients with advanced hematologic malignancies, durable responses were observed, with neutropenia (46%) and thrombocytopenia (36%) being the most common treatment-related adverse events [192]. A subsequent phase II study in patients with aggressive NHL is ongoing (NCT00807495).

The selective ABK inhibitor, AZD1152, potently inhibited a range of tumor xenografts in immunodeficient mice [193] and is currently in phase I/II development for DLBCL (NCT01354392). Aurora kinases in preclinical development include the novel pan-Aurora/JAK-2 kinase inhibitor AT9283 [194].

A number of cyclin modulators are currently in development, including the cyclin-dependant kinase (CDK) inhibitors flavopiridol, which is in a phase I/II study in relapsed MCL/DLBCL (NCT00445341), and dinaciclib (SCH 727965), which has shown clinical responses in a phase I study in heavily pretreated diffuse large cell lymphoma [195]. A phase I dose-escalation study of the cyclin D modulator ON 013105 in patients with R/R lymphoma is ongoing (NCT01049113) after showing promising *in vitro* and *in vivo* data in MCL [196].

Fostamatinib is a spleen tyrosine kinase (Syk) inhibitor which has shown synergistic activity with a number of agents in *in vivo* models of DLBCL [197]. In a recent phase I/II study in NHL and CLL, substantial responses were observed in a number of tumor types. Common toxicities included diarrhea, fatigue, cytopenias, and hypertension [198].

Activation of protein kinase C (PKC) and its overexpression have been associated with a less favorable outcome in DLBCL [199]. Enzastaurin is an inhibitor of PKC-*β* [200]. In a phase II study in R/R DLBCL, prolonged freedom from progression (FFP) was observed with little grade 3 toxicity. Preliminary results from a subsequent study in aggressive NHL also indicate single-agent activity [201]. A phase III study with daily enzastaurin to prevent relapse in DLBCL patients in remission after R-CHOP treatment is currently ongoing (NCT00332202).

Dasatinib has shown single-agent activity in a phase I/II study in R/R NHL [202]. Pleural effusions and cytopenias were the main grade 3 or 4 toxicities. A phase II study in R/R DLBCL (NCT00918463) is currently recruiting.

Bruton's tyrosine kinase (Btk) is a mediator of B-cell signaling, and PCI-32765 is a selective, irreversible inhibitor of Btk [203]. In a phase I study in patients with R/R B-cell malignancies, PCI-32765 induced durable responses with minimal toxicity [204]. Encouraging initial clinical results with the anaplastic lymphoma kinase (ALK) inhibitor crizotinib in advanced chemoresistant ALK^+^ lymphoma patients have also been observed [205].

The benzimidazole AZD6244 (ARRY-142886) is a novel, 2nd-generation mitogen-activated protein kinase (MEK) inhibitor [206]. Considerable cell death was shown in DLBCL cell lines, primary cells, and in an *in vivo* xenograft model, at clinically achievable concentrations.

### 5.7. JAK/STAT Pathway

The Janus kinase 2 (JAK-2)/signal transducers and activators of transcription (STAT) pathway play a key role in the proliferation and pathogenesis of hematologic malignancies [207]. A phase I study of the novel JAK-2 inhibitor, SB1518, has provided evidence of activity in patients with relapsed lymphoma. Degrasyn, a novel, small-molecule inhibitor of the JAK/STAT pathway, has been shown to interact synergistically with bortezomib *in vivo* to prevent tumor development and to prolong survival time in a xenotransplant severe combined immunodeficient (SCID) mouse model of MCL [208].

### 5.8. Toll-Like Receptor (TLR) Agonist (Table 5)

PF-3512676 is a novel TLR9-activating oligonucleotide with single-agent antitumor activity that augments preclinical rituximab efficacy [209]. Preliminary antitumor activity for the combination was found by a phase I study in patients with recurrent, indolent, and aggressive NHL, while grade 3 or 4 neutropenia occurred in 4/50 patients. 

Evaluation of a combination regimen involving a TLR7/8 dual agonist (IMO-4200) with rituximab, bortezomib, or cyclophosphamide, in human xenograft and murine syngeneic lymphoma models suggests that the antitumor activity of these agents in the treatment of NHL and other hematologic malignancies could be enhanced using this strategy [210]. The transforming-growth-factor- (TGF-) *β*-activated kinase 1 (TAK-1) inhibitor, AZ-Tak1, has been shown to inhibit X-linked inhibitor of apoptosis protein (XIAP), activate caspase-9, and induce apoptosis in MCL cell lines [211].

Immunostimulatory CpG oligodeoxynucleotides (ODNs) are potent activators of T-cell immunity and antibody-dependent cellular cytotoxicity (ADCC) and are under investigation as immunotherapeutic agents for a variety of malignancies, including BCL [212]. Anti-CD20 antibody-CpG conjugates have been shown to eradicate rituximab-resistant BCL in a syngeneic murine lymphoma model. A recent demonstration of the divergent effects of CpG ODNs on normal versus malignant B cells may suggest a novel mechanism of action for CpG ODNs as therapeutic agents for BCL [213].

### 5.9. Heat Shock Proteins (Hsps) (Table 5)

Hsps are chaperones needed for the correct functioning of proteins involved in cell growth and survival [214]. Inhibition of these proteins results in increased degradation of key proteins such as kinases, signal transducer proteins, and mutated oncogenic proteins. GUT-70, a tricyclic coumarin derived from *Calophyllum brasiliense*, has shown pronounced antiproliferative effects in MCL with mutant-type *p53* (*mt-p53*), a known negative prognostic factor for MCL, through Hsp90 inhibition. These findings suggest that GUT-70 could be potentially useful for the treatment of MCL [215].

The small-molecule 17-AAG (17-allylamino-17-demethoxygeldanamycin) can induce cell death in a dose- and time-dependent manner by reducing the cellular contents of critical survival proteins, including Akt and cyclin D1 in a range of lymphoma cell lines [216]. Several clinical responses were observed in a phase II study of 17-AAG in patients with R/R MCL or HL. SNX-2112 was found to exert effects in combination with bortezomib and rituximab in rituximab-resistant NHL cell lines [181]. SNX-2112 is currently in phase I clinical trials.

### 5.10. Angiogenesis (Table 5)

Tumor angiogenesis is important in a variety of hematologic malignancies [217]. Bevacizumab, already widely studied in solid tumors, has also been evaluated in lymphoma. In a phase II SWOG study of R-CHOP plus bevacizumab in patients with advanced DLBCL, the observed 1-year PFS estimate trended higher than the historical estimate. However, as significant toxicities were associated with the addition of bevacizumab the regimen was not recommended for further evaluation [218]. In a phase II study of single-agent sunitinib in R/R DLBCL, no evidence of activity was recorded and hematologic toxicities were greater than anticipated [219]. The vascular-endothelial-growth-factor- (VEGF-) 1/2 fusion protein, aflibercept, has been evaluated in a phase I study in combination with R-CHOP in untreated patients with BCLs [220]. The 6 mg/kg dose of aflibercept is used in all ongoing phase III trials in other indications, and the combination with R-CHOP resulted in high response rates in this study. The main grade 3 or 4 adverse events included hypertension (8/25), febrile neutropenia (4/25), and asthenia (4/25). 

Preliminary results are available from 2 recent phase II trials with sorafenib. In a single-agent study in heavily pretreated patients with R/R NHL, a number of responses were noted and therapy was overall well tolerated [221]. In a phase II study in combination with the Akt inhibitor perifosine in R/R lymphomas, a number of PRs were observed, with thrombocytopenia (18%) the most common drug-related hematological toxicity [222]. A phase II study in recurrent DLBCL is currently ongoing (NCT00131937). The combination of sorafenib and everolimus was shown to be well tolerated, with activity observed, especially in HL, in a phase I trial in patients with lymphoma or MM [223].

### 5.11. Additional Targeted Agents and Novel Therapeutics

Farnesyltransferases are key cellular enzymes involved in the prenylation of proteins [224]. Prenylated proteins are important for malignant cell growth. The oral farnesyltransferase inhibitor, tipifarnib, has been assessed in a phase II study in patients with relapsed, aggressive, indolent, or uncommon lymphoma. Tipifarnib had a good tolerability profile and demonstrated activity in lymphoma, with responses in patients with heavily pretreated DLBCL, HL, and T-cell types, although little activity was observed in follicular NHL.

MLN4924 is an investigational inhibitor of Nedd8-activating enzyme (NAE), which plays a crucial role in regulating the activity of the cullin-RING E3 ligases (CRLs) [225]. Preclinical activity has been demonstrated in a novel primary human DLBCL xenograft model [226] and a phase 1 dose-escalation study of multiple dosing schedules is currently underway in patients with R/R MM or lymphoma [225].

Potential molecular targets for novel therapeutics (or “theranostics”) are beginning to be identified through an emerging area in lymphoma biology involving energy metabolism. Personalized medicine approaches using bifunctional imaging and therapeutic agents are based on the premise that glucose metabolism rates are high in aggressive B-cell lymphomas [227]. Use of this bifunctional pathway as a targeted therapy has been explored recently with ^187^rhenium-ethylenedicysteine-N-acetylglucosamine, a synthetic glucose analog, which accumulates in cancer cell nuclei and in various tumors in animal models. Biodistribution data revealed that radioactivity was retained in tumor tissue 2 hours after injection with little uptake in the plasma when compared with tumor tissue. The compound was excreted over a longer incubation period, and the retention time in lymphoma tissue was longer than that of other tissues. The results suggest that the metallic pharmaceutical agent 187Re-ECG may be a potential candidate for targeted therapy in aggressive R/R lymphomas.

The recently developed, small-molecule MDM2 antagonist, nutlin-3, inhibits the MDM2-p53 interaction, resulting in stimulation of p53 activity and apoptosis [228]. The cytotoxic effects of nutlin-3 on ALL cells suggest that the agent may be a novel therapeutic for refractory ALL [138, 228].

Stromal-cell-derived-factor-1 (SDF-1) is a chemokine that binds to the CXCR4 chemokine receptor and stimulates B-cell growth [229]. CXCR4 is frequently overexpressed on tumor cells, and the SDF-1/CXCR4 axis is thought to play a role in promoting survival, angiogenesis, and metastasis. Treatment with the CXCR4 antagonist, AMD3100, has been shown to enhance antibody-mediated cell death in disseminated lymphoma models, suggesting a potential role for CXCR4 antagonists in combination with a B-cell targeted therapy in the treatment of B-cell malignancies in the clinical setting.

MCL is characterized by the translocation t(11; 14)(q13; q32) [230]. All-trans retinoic acid (ATRA) is a key retinoid that acts through nuclear receptors that function as ligand-inducible transcription factors [231]. MCL cells express retinoid receptors; therefore ATRA may exert antiproliferative effects and, thus, may have a role in treatment. In a recent study, a novel approach to deliver ATRA to MCL cells in culture involved stably incorporating the water-insoluble bioactive lipid into nanoscale lipid particles, termed nanodisks (ND), comprised of disk-shaped phospholipid bilayers stabilized by amphipathic apolipoproteins. ATRA-ND was shown to enhance apoptosis and cell-cycle arrest in MCL cell lines, resulting in increased p21, p27, and p53 expression and decreased cyclin D1 expression; these results suggest that ATRA-ND may represent a potentially effective approach to the treatment of MCL.

Hypoxia-inducible-factor-1 (HIF-1) is a transcription factor that serves as a master regulator of cellular responses to hypoxia and regulates genes required for adaptation to hypoxic conditions [232]. HIF-1a is commonly activated in cancer cells, including under normoxic circumstances, by oncogene products or by impaired activity of tumor suppressor genes. PX-478, the novel, small-molecule HIF-1a inhibitor, has been shown to downregulate HIF-1a protein at low concentrations effectively and to induce cell death in DLBCL cells.

## 6. Conclusion

In addition to the numerous cytotoxic combination regimens already available, a myriad of new agents are in development, targeting key molecular pathways critical to aggressive B-cell growth (Table 7). As monotherapy, or in combination with chemotherapy or other targeted agents, these new pharmacotherapies are likely to provide additional clinical benefit to patients with aggressive B-cell NHL and represent continued progress in the search for individualized treatments. As individualized therapy will depend on the identification of predictive markers, future clinical trials should incorporate the identification of molecular markers in their “smart” trial design. How the search for individualized treatment will affect drug development and improve clinical trial design remains to be seen.

## Figures and Tables

**Table 1:** Cytotoxic therapies in clinical development for the treatment of aggressive NHL. [B: bendamustine; CLL: chronic lymphocytic leukemia; CR: complete response; CRu: unconfirmed CR; DLBCL: diffuse large B-cell lymphoma; EFS: event-free survival; F: fludarabine; HL: Hodgkin lymphoma; MCL: mantle cell lymphoma; MOA: mechanism of action; mOS: median overall survival; mPFS: median progression-free survival; NHL: non-Hodgkin lymphoma; ORR: overall response rate; OS: overall survival; P: pixantrone; PFS: progression-free survival; PR: partial response; R: rituximab; R-CHOP: cyclophosphamide, doxorubicin, vincristine, prednisolone plus rituximab; R/R: relapsed or refractory; SD: stable disease.]

Drug	MOA (target)	Eligibility (and design)	Phase	Randomized	Results
Bendamustine [46]	Alkylating agent	R/R NHL/CLL	Registry	No	ORR: 84.6% in MCL
Bendamustine [45]	Alkylating agent	R/R Lymphoma (±R)	Retrospective analysis	No	MCL: ORR: 67%; 1-year OS: 68%; 1-year PFS: 15% DLBCL: ORR: 31%; 1-year OS: 27%; 1-year PFS: 10%
Bendamustine [42]	Alkylating agent	R/R DLBCL (+R)		No	ORR: 51.6%
Bendamustine [40]	Alkylating agent	R/R aggressive NHL	I	No	B 90 mg/m^2^: ORR: 33%;B 120 mg/m^2^: ORR: 100%
Bendamustine [41]	Alkylating agent	R/R MCL/NHL	II	No	ORR: 100% in MCL
Bendamustine [44]	Alkylating agent	Previously untreated follicular + indolent + MCL (with R versus R-CHOP)	III	Yes	BR versus R-CHOP: CR: 40.1% versus 30.8% (*P* = .0323); PFS: 54.8 months versus 34.8 months (*P* = .0002); OS: no difference
Bendamustine [43]	Alkylating agent	Follicular + indolent + MCL with R versus FR	III	Yes	BR versus FR: CR: 83.5% versus 52.5% (*P* < .0001); PFS: 30 months versus 11 months (*P* < .0001); OS: no difference
Pixantrone [49]	Aza-anthracenedione	R/R aggressive NHL (versus other single agents)	III	Yes	*P* versus comparator: ORR: 37% versus 14%; CR/CRu: 20% versus 6%; PFS: 4.7 versus 2.6 months; mPFS (*P* = .007); mOS: 8.1 versus 6.9 months (HR = 0.88, *P* = .554)
SB-743921 [51]	Kinesin spindle protein inhibitor	R/R HL or NHL	I/II dose-finding study	No	4 PRs: 3 in HL, 1 in marginal-zone NHL; 1 durable SD (>17 months) in DLBCL
Gemcitabine/oxaliplatin [45]	Chemotherapy	R/R DLBCL with rituximab	II	No	ORR: 43%; CR: 34%; 12-month PFS rate: 29%; 12-month OS rate: 41%
Gemcitabine/oxaliplatin [46]	Chemotherapy	R/R MCL with rituximab		No	CR/CRu: 77%; 2-year PFS rate: 41%; 2-year OS rate: 58%
Gemcitabine/oxaliplatin [47]	Chemotherapy	R/R B-cell NHL with rituximab	II	No	ORR: 83%; CR/CRu: 50%; 2-year EFS: 42%; 2-year OS: 66%
Gemcitabine/oxaliplatin [48]	Chemotherapy	R/R B-cell NHL (transplant ineligible) with R		No	GemOx: ORR: 57%; CR: 30% R-GemOx: ORR: 78%; CR: 50%
Liposomal vincristine [50]	Chemotherapy	R/R NHL	II	No	ORR: 25%; CR/CRu: 5%

**Table 2:** Therapeutic antibodies in clinical development for the treatment of aggressive NHL. [CR: complete response; CRu: unconfirmed CR; DLBCL: diffuse large B-cell lymphoma; EFS: event-free survival; EOTR: extent of tumor resection; FL: follicular lymphoma; HL: Hodgkin lymphoma; mAb: monoclonal antibody; MCL: mantle cell lymphoma; mDR: median duration of response; MOA: mechanism of action; mPFS: median progression-free survival; MZL: marginal zone B-cell lymphoma; NHL: non-Hodgkin lymphoma; ORR: overall response rate; OS: overall survival; PFS: progression-free survival; PR: partial response; R-CHOP: cyclophosphamide, doxorubicin, vincristine, prednisolone plus rituximab; R: rituximab; R/R: relapsed or refractory; RR: response rate.]

Drug	MOA (target)	Eligibility (and design)	Phase	Randomized	Results
Ofatumumab [60]	Anti-CD20 mAb	R/R DLBCL	II	No	ORR: 11%; CR: 4%; mDR: 6.9 months; mPFS: 2.5 months
GA101 [67]	Anti-CD20 mAb	R/R DLBCL and MCL	II	Yes	EOTR: All: 28%; DLBCL: 29%; MCL: 27%
Veltuzumab [56]	Anti-CD20 mAb	R/R NHL	I/II	No	ORR: DLBCL: 43%; MZL: 83%, including CR/CRu: 33% ORR: FL: 44%, including CR/CRu: 27%
Epratuzumab [69]	Anti-CD22 mAb	R/R NHL (with rituximab)	II	No	ORR: 47%; DLBCL: CR: 33%
Epratuzumab [70]	Anti-CD22 mAb	Previously untreated DLBCL (with R-CHOP)	II	No	ORR: 95%; CR/CRu: 73%; 1-year EFS rate: 80%; 1-year PFS rate: 82%; 1-year OS rate: 88%
Milatuzumab [57]	Anti-CD74 mAb	R/R NHL (with veltuzumab)	I/II	Dose-finding	PR: 1/3 in Cohort 1 (8 mg/kg); 2/3 in Cohort 2 (16 mg/kg)
Dacetuzumab [77]	Anti-CD40 mAb	R/R DLBCL (with rituximab and gemcitabine)	Ib	Dose-finding	ORR: 54%
Lucatumumab [74]	Anti-CD40 mAb	R/R HL or NHL	Ia/II	Dose-finding	RR: R refractory: 40%; ORR: DLBCL: 11% (phase Ia); ORR: DLBCL: 15% (phase II)
Blinatumomab (MT103) [83]	Single-chain bispecific anti-CD19 and CD3 mAb construct	R/R NHL	I	Dose-finding	FL: 11/21 responses; MCL: 3/21 responses

**Table 3:** Antibody-drug conjugates and radiolabeled antibodies in clinical development for the treatment of aggressive NHL. [CR: complete response; CRu: unconfirmed CR; DLBCL: diffuse large B-cell lymphoma; HDT-ASCT: high-dose therapy/autologous stem cell transplantation; mAb: monoclonal antibody; MOA: mechanism of action; mPFS: median progression-free survival; NHL: non-Hodgkin lymphoma; ORR: overall response rate; OS: overall survival; PFS: progression-free survival; R: rituximab; R-CHOP: cyclophosphamide, doxorubicin, vincristine, prednisolone plus rituximab; RIT: radioimmunotherapy; R/R: relapsed or refractory.]

Drug	MOA (target)	Eligibility (and design)	Phase	Randomized	Results
I-131 tositumomab [96]	Anti-CD20 radioimmunotherapy	Previously untreated DLBCL (with R-CHOP)		No	1-year PFS rate: 75%; 1-year OS rate: 83%
Inotuzumab ozogamicin (CMC-544) [93]	CD22 targeted cytotoxic immunoconjugate	R/R CD22^+^ and CD20^+^ NHL (with R)	I	No	ORR: 80%; 1-year PFS rate: 89%
Inotuzumab ozogamicin (CMC-544) [94]	CD22 targeted cytotoxic immunoconjugate	R/R CD22^+^ and CD20^+^ DLBCL prior to HDT-ASCT (with R)		No	ORR: 21%
^90^Y-epratuzumab tetraxetan [92]	Radiolabeled humanized anti-CD22 mAb	R/R NHL	I/II	Dose-finding	ORR: 62%; CR/CRu: 48%; mPFS: 9.5 months
^90^Y-epratuzumab tetraxetan [97]	Radiolabeled humanized anti-CD22 mAb	Consolidation after first-line R-CHOP in DLBCL	II	No	Improved remission status 6 weeks after RIT: 30.7%
Brentuximab vedotin (SGN-35) [104]	Antitubulin monomethyl auristatin E (MMAE) anti-CD30 mAb conjugate	R/R lymphoma	I		ORR: 46%; CR: 29%

**Table 4:** Immunomodulatory agents in clinical development for the treatment of aggressive NHL. [B: bortezomib; CR: complete response; CRR: complete response rate; CRu: unconfirmed CR; Dex: dexamethasone; MCL: mantle cell lymphoma; mDR: median duration of response; mEFS: median event-free survival; MOA: mechanism of action; mPFS: median progression-free survival; NHL: non-Hodgkin lymphoma; ORR: overall response rate; OS: overall survival; PR: partial response; R: rituximab; R-CHOP21: cyclophosphamide, doxorubicin, vincristine, prednisolone plus rituximab, every 21 days; R/R: relapsed or refractory; TTF: time to treatment failure.]

Drug	MOA (target)	Eligibility	Phase	Randomized	Results
Thalidomide [111]	Immunomodulator	R/R MCL (alone, with R, with B, with RB, with other agents)	NA	No	ORR: 50%; CR: 20.7; 2-year TTF rate: 10.9%; 2-year OS rate: 49.6%
Lenalidomide [113]	Immunomodulator	R/R aggressive NHL	II	No	ORR: 35%; CR/CRu: 12%; mDR: 6.2 months; mPFS: 4.0 months
Lenalidomide [114]	Immunomodulator	R/R aggressive NHL	II	No	ORR: 35%; CR/CRu: 13%; mPFS: 3.5 months
Lenalidomide [116]	Immunomodulator	R/R MCL	II	No	ORR: 53%; CR: 20%; mPFS: 5.6 months
Lenalidomide [117]	Immunomodulator	R/R MCL (with Dex)	II	No	ORR: 55%; CR: 24%
Lenalidomide [118]	Immunomodulator	R/R MCL (with R)	I/II	Dose-finding	ORR: 53%; CRR: 31%; mPFS: 14.0 months
Lenalidomide [120]	Immunomodulator	R/R Indolent or MCL (with R/Dex)	II	No	ORR: 57%; mEFS: 12.0 months; 78%
Lenalidomide [121]	Immunomodulator	Previously untreated DLBCL (with R-CHOP21)	I/II	Dose-finding	CR: 15/21; PR: 1/21

**Table 5:** Targeted therapies in clinical development for the treatment of aggressive NHL. [ABC: activated B-cell-like DLBCL; CR: complete response; CRu: unconfirmed CR; DLBCL: diffuse large B-cell lymphoma; FL: follicular lymphoma; GCB: germinal-center B-cell-like DLBCL; HDACI: histone deacetylase inhibitor; HL: Hodgkin lymphoma; Hsp: heat shock protein; mAB: monoclonal antibody; MCL: mantle cell lymphoma; mDR: median duration of response; MOA: mechanism of action; mOS: median overall survival; mPFS: median progression-free survival; mTOR: mammalian target of rapamycin; MZL: marginal zone B-cell lymphoma; NHL: non-Hodgkin lymphoma; ORR: overall response rate; OS: overall survival; PI3K: phosphatidylinositol 3-kinase; PFS: progression-free survival; PR: partial response; R: rituximab; R-CHOP: cyclophosphamide, doxorubicin, vincristine, prednisolone plus rituximab; R-ICE: rituximab, ifosfamide, carboplatin, and etoposide; RiPAD+C: bortezomib plus rituximab, doxorubicin, dexamethasone, chlorambucil; R/R: relapsed or refractory; TLR: Toll-like receptor; VEGF: vascular endothelial growth factor; VEGFR: vascular endothelial growth factor receptor.]

Drug	MOA (target)	Eligibility	Phase	Randomized	Results
Bortezomib [127]	Proteasome inhibitor	Previously untreated DLBCL (with R-CHOP)	I/II	Dose-finding	CR/CRu: 92%
Bortezomib [128]	Proteasome inhibitor	R/R DLBCL (with chemotherapy)	II	No	ABC versus GCB: ORR: 83% versus 13%; mOS: 10.8 versus 3.4 months
Bortezomib [130]	Proteasome inhibitor	R/R indolent nonfollicular + MCL (+R)	II	No	ORR: 53%; CR: 26.5%; PR: 26.5% 2-year OS: 80%; 2-year PFS: 25%
Bortezomib [126]	Proteasome inhibitor	R/R indolent + MCL with R/bendamustine	II	No	ORR: 84%; CR/CRu: 52%
Bortezomib [131]	Proteasome inhibitor	Previously untreated MCL (RiPAD + C)	II	No	ORR: 80%; CR: 51% after 4 cycles
NPI-0052 [141]	Proteasome inhibitor	Multiple tumor types	I/II	Dose-finding	Clinical benefit observed in multiple tumor types including MCL, HL, cutaneous MZL, and FL
Everolimus [150]	mTOR inhibitor	R/R MCL	II	No	ORR: 12%
Everolimus [151]	mTOR inhibitor	R/R NHL	II	No	ORR: 30%; mDR: 5.7 months
Everolimus [152]	mTOR inhibitor	R/R NHL	I	2 dose cohorts	2 responses in DLBCL and 2 responses in FL, in 13 patients
Everolimus [149]	mTOR inhibitor	R/R MCL	II	No	ORR: 20%; mDR: 5.45 months
Temsirolimus [153]	mTOR inhibitor	R/R MCL (2 doses, compared with investigators' choice therapy)	III	Yes	ORR: 22% (temsirolimus 175/75 mg) versus 2% (investigators' choice); mPFS: 4.8 months (temsirolimus 175/75 mg) versus 3.4 months (temsirolimus 175/25 mg) versus 1.9 months (investigators' choice); OS: 12.8 months (temsirolimus 175/75 mg) versus 9.7 months (investigator's choice)
Temsirolimus [154]	mTOR inhibitor	R/R MCL (with rituximab)	II	No	ORR: 59%; CR: 19%; PR: 40%
Vorinostat [167]	Deacetylase inhibitor	R/R lymphoma (with R-ICE)	I	No	19/27 responses
Vorinostat [168]	Deacetylase inhibitor	R/R lymphoma (with DOXIL)	I	Dose-finding	4/14 disease control
Oblimersen sodium [183]	Bcl-2 antisense oligonucleotide	R/R B-cell NHL (with R)	II	No	ORR: 42%; ORR in FL: 60%
PF-3512676 [209]	TLR9-antagonist	R/R NHL (with R)	I	Dose-finding	ORR: 24%; ORR in extended treatment cohort: 50%
17-AAG [216]	HSP90 inhibitor	R/R MCL or HL	II	No	ORR: 11% (all PR)
Bevacizumab [221]	Anti-VEGF mAb	Previously untreated DLBCL (with R-CHOP)	II	No	1-year PFS rate: 77%; 2-year PFS rate: 69%; 1-year OS rate: 86%; 2-year OS rate: 79%
Aflibercept [220]	VEGF fusion protein	Previously untreated B-cell lymphoma (with R-CHOP)	I	Dose-finding	ORR: 100%; CR: 80%
CAL-101 [157]	PI3K inhibitor	R/R NHL	I	No	RR: relapsed MCL: 73%;RR: refractory MCL: 40%
Valproic acid [171]	HDACI	R/R NHL	II	No	ORR: 29% (all PR)

**Table 6:** Kinase inhibitors currently in clinical development for the treatment of aggressive NHL. [CDK: cyclin-dependant kinase; CLL: chronic lymphocytic leukemia; CR: complete response; DLBCL: diffuse large B-cell lymphoma; ERK: extracellular signal regulated kinase; FFP: freedom from progression; FL: follicular lymphoma; HL: Hodgkin lymphoma; JAK: Janus kinase; MCL: mantle cell lymphoma; mPFS: median progression-free survival; NHL: non-Hodgkin lymphoma; MEK: mitogen-activated protein kinase kinase; MM: multiple myeloma; mTOR: mammalian target of rapamycin; ORR: overall response rate; OS: overall survival; PDGF: platelet-derived growth factor; PDGFR: platelet-derived growth factor receptor; PFS: progression-free survival; PR: partial response; R/R: relapsed or refractory; RTK: receptor tyrosine kinase; SD: stable disease; SLL: small lymphocytic lymphoma; TKI: tyrosine kinase inhibitor; VEGFR: vascular endothelial growth factor receptor.]

Drug	MOA (target)	Eligibility	Phase	Randomized	Results
Dinaciclib [183]	CDK1, 2, 5, 9 inhibitor	R/R in low-grade lymphoma and DLBCL	I	No	PR: DLBCL: 1/7; SD: FL: 2/7; MCL: 1/1
Fostamatinib [198]	Syk inhibitor	R/R B-cell NHL and CLL	I/II	No	ORR: DLBCL: 22%; FL: 10% SLL/CLL: 55% in SLL/CLL: MCL: 11%; overall mPFS: 4.2 months
Dasatinib [202]	RTK inhibitor of BCR-ABL, SRC, c-Kit, PDGF and ephrin receptor kinases	R/R NHL	I/II	No	ORR: 32%; 2-year PFS: 13%; 2-year OS: 50%
Enzastaurin [201]	Protein kinase beta inhibitor	R/R DLBCL	II	No	FFP: 22%
PCI-32765 [204]	Bruton's tyrosine kinase inhibitor	R/R B-cell NHL	I	Dose-finding	ORR: 43%
SB1518 [208]	JAK2 inhibitor	R/R lymphoma	I	Dose-finding	PR: 3/26 (2 in MCL)
Sorafenib [221]	TKI inhibitor of RAF/MEK/ERK/c-kit/Flt3, VEGFRs, PDGFRs, RET	R/R NHL	II	No	ORR: 10%; CR: 5%
Sorafenib [222]	TKI inhibitor of RAF/MEK/ERK/c-kit/Flt3, VEGFRs, PDGFRs, RET	R/R lymphoma (with Akt inhibitor perifosine)	II	No	ORR: 23% (all PR; all in HL)
Sorafenib [223]	TKI inhibitor of RAF/MEK/ERK/c-kit/Flt3, VEGFRs, PDGFRs, RET	R/R MM or lymphoma (with mTOR inhibitor everolimus)	I/II	Dose-finding	ORR: 33%

**Table 7:** Overview of ongoing or recently completed phase III studies mentioned in this paper, with agents in clinical development for the treatment of aggressive NHL. [B: bendamustine; CR: complete response; DLBCL: diffuse large B-cell lymphoma; F: fludarabine; MCL: mantle cell lymphoma; NA: not applicable; NHL: non-Hodgkin lymphoma; PFS: progression-free survival; R-CHOP: cyclophosphamide, doxorubicin, vincristine, prednisolone plus rituximab; R: rituximab; R/R: relapsed or refractory; SCT: stem cell transplantation.]

Drug	Indication	Study identifier	Study status	Results
Enzastaurin	DLBCL in remission after R-CHOP treatment	NCT00332202 PRELUDE	Ongoing; not recruiting	NA
				
Inotuzumab ozogamicin + R versus investigator's choice of gemcitabine + R or B + R	R/R aggressive NHL	NCT01232556	Recruiting	NA
				
B + R versus F + R [41]	R/R follicular, indolent, and MCL	NCT01456351	Completed; final results presented at ASH 10	B + R had better efficacy than F + R
B + R versus R-CHOP [42]	Previously untreated follicular, indolent, and MCL	NCT00991211	Completed; final results presented at ASH 09	B + R superior to R-CHOP for CR and PFS
				
Single-agent pixantrone dimaleate versus investigator's choice therapy [49]	Third-line treatment of R/R aggressive NHL	NCT00088530 EXTEND (PIX301)	Completed; final results presented at ASH 10	Pixantrone superior to other single-agent therapies
Pixantrone + R versus gemcitabine + R	R/R DLBCL patients not eligible for SCT	NCT01321541 PIX-R (PIX306)	Recruiting	NA
